# Evaluation of Skin Recovery after Fractional CO_2_ Laser Treatment Lichen Sclerosus Using Multimodal Optical Coherence Tomography

**DOI:** 10.17691/stm2024.16.4.02

**Published:** 2024-08-30

**Authors:** M.A. Sirotkina, A.L Potapov, M.M. Loginova, A.E. Bychkova, A.A. Moiseev, M.V. Kochuyeva, A.Yu. Bogomolova, E.B. Kiseleva, A.V. Asaturova, A.V. Maslennikova, S.G. Radenska-Lopovok, LA. Apolikhina, N.D. Gladkova

**Affiliations:** PhD, Director, Research Institute of Experimental Oncology and Biomedical Technologies; Privolzhsky Research Medical University, 10/1 Minin and Pozharsky Square, Nizhny Novgorod, 603005, Russia; Laboratory Assistant, Laboratory of Optical Coherence Tomography, Research Institute of Experimental Oncology and Biomedical Technologies; Privolzhsky Research Medical University, 10/1 Minin and Pozharsky Square, Nizhny Novgorod, 603005, Russia; PhD, Junior Researcher, Laboratory of Optical Coherence Tomography, Research Institute of Experimental Oncology and Biomedical Technologies; Privolzhsky Research Medical University, 10/1 Minin and Pozharsky Square, Nizhny Novgorod, 603005, Russia; Obstetrician-Gynecologist, Esthetic Gynecology and Rehabilitation Unit; National Medical Research Center for Obstetrics, Gynecology, and Perinatology named after Academician V.I. Kulakov of the Russian Ministry of Health, 4 Academician Oparin St., Moscow, 117997, Russia; PhD, Senior Researcher, Laboratory of High-Sensitivity Optical Measurements; Federal Research Center Institute of Applied Physics of the Russian Academy of Sciences, 46 Ulyanova St., Nizhny Novgorod, 603950, Russia; Oncologist, Head of the Center for Outpatient Oncological Aid, MEDSI Clinic in Otradnoye; MEDSI Group of Companies, 3a Gruzinsky Alley, Moscow, 123056, Russia; Laboratory Assistant, Laboratory of Regenerative Medicine, Research Institute of Experimental Oncology and Biomedical Technologies; Privolzhsky Research Medical University, 10/1 Minin and Pozharsky Square, Nizhny Novgorod, 603005, Russia; PhD, Senior Researcher, Laboratory of Optic Coherence Tomography, Research Institute of Experimental Oncology and Biomedical Technologies; Privolzhsky Research Medical University, 10/1 Minin and Pozharsky Square, Nizhny Novgorod, 603005, Russia; MD, DSc, Head of the 1^st^ Department of Pathology; National Medical Research Center for Obstetrics, Gynecology, and Perinatology named after Academician V.I. Kulakov of the Russian Ministry of Health, 4 Academician Oparin St., Moscow, 117997, Russia; MD, DSc, Professor, Head of the Department of Oncology, Radiotherapy and Diagnostic Radiology; Privolzhsky Research Medical University, 10/1 Minin and Pozharsky Square, Nizhny Novgorod, 603005, Russia; Radiotherapist; Nizhny Novgorod Regional Oncologic Dispensary, 11/1 Delovaya St., Nizhny Novgorod, 603093, Russia; MD, DSc, Professor, Institute of Clinical Morphology and Digital Pathology; I.M. Sechenov First Moscow State Medical University (Sechenov University), 8/2 Trubetskaya St., Moscow, 119991, Russia; MD, DSc, Professor, Head of the Esthetic Gynecology and Rehabilitation Unit; National Medical Research Center for Obstetrics, Gynecology, and Perinatology named after Academician V.I. Kulakov of the Russian Ministry of Health, 4 Academician Oparin St., Moscow, 117997, Russia; Professor, Department of Obstetrics, Gynecology, Perinatology, and Reproductology; I.M. Sechenov First Moscow State Medical University (Sechenov University), 8/2 Trubetskaya St., Moscow, 119991, Russia; MD, DSc, Professor, Head of the Laboratory of Optical Coherence Tomography Research Institute of Experimental Oncology and Biomedical Technologies; Privolzhsky Research Medical University, 10/1 Minin and Pozharsky Square, Nizhny Novgorod, 603005, Russia

**Keywords:** vulvar lichen sclerosis, fractional CO_2_ laser, multimodal optical coherence tomography, MM OCT, OCT signal attenuation coefficient, angiography, lymphangiography

## Abstract

**The aim of the study** is to demonstrate the effect of fractional CO_2_ laser treatment on vulvar skin in lichen sclerosus using multimodal optical coherence tomography (MM OCT).

**Materials and Methods:**

The study included 3 clinical cases of vulvar lichen sclerosus (VLS) (histologically classified as early, early with dermal edema, late) and 2 control cases without vulvar pathology. Patients with VLS underwent 3 procedures of fractional CO_2_ laser treatment with an interval of 30-40 days. In patients without vulvar pathology, the MM OCT examination was performed once, in patients with VLS it was done at the point of the greatest visible skin damage just before a punch biopsy, immediately after the first laser session, and 1 month after a full course of treatment.

**Results:**

Analyzing the case series of vulvar skin recovery 1 month after fractional CO_2_ laser treatment, it has been found that the recovery depended on the dermal condition before the treatment. In early VLS and early VLS with dermal edema (clinical cases 1 and 2) before the treatment, the MM OCT examination has shown a decreased epidermal thickness, unclear contrast between the epidermis and dermis, a decrease in the values of the OCT signal attenuation coefficient in the dermis, and a sharp reduction in the density of blood and lymphatic vessels. After treatment, the MM OCT examination demonstrated a complete recovery of vulvar skin structure and all quantitative values reached the level characteristic of normal vulvar skin. Before the treatment, a homogenization zone (sclerosis) was clearly visible in the MM OCT images of the dermis in late VLS (clinical case 3). After the treatment, complete recovery of the vulvar skin structure was not observed; the area of sclerosis was preserved; however, visually, there was an increase in the density of blood and lymphatic vessels in the affected area, which almost reached the level of normal vulvar skin.

**Conclusion:**

MM OCT can be proposed as a promising non-invasive method for monitoring skin recovery after fractional CO_2_ laser treatment of VLS.

## Introduction

Optical coherence tomography (OCT) is a valuable non-invasive method of real time optical tissue visualization to the depth of 2 mm. OCT is functioning as a sort of “optical biopsy” in the near-infrared range and allows for visualization of a tissue microstructure with the resolution close to the histological one. Multiple investigations have demonstrated a wide spectrum of OCT applications in gynecology [1], however, recently there has emerged a new topical area connected with diagnosing vulvar and vaginal diseases such as vulvar intraepithelial neoplasia [2], vulvar dermatoses [2, 3], genitourinary menopausal syndrome [4, 5], vaginal wall prolapse [6], and with monitoring the treatment of these pathologies [5]. OCT has some advantages over the histological investigation such as non-invasiveness allowing for intravital investigations of the tissue structure, obtaining information in real time, exploration of multiple areas, and evaluation of the results in dynamics. Multimodal OCT (MM OCT) gives the possibility to receive information on the condition of epithelium, dermis, blood, and lymphatic vessels simultaneously.

Vulvar lichen sclerosus (VLS) is a chronic progressing immune-mediated inflammatory disease of the vulvar skin demanding a long-term treatment and remission maintenance [7]. VLS manifests itself by debilitating symptoms (itching, pain in the vulvar area, dyspareunia, difficulty in urination, reduced sexual activity, formation of fissures and fusions), which reduces women’s quality of life [7, 8]. VLS is a predisposing factor for developing vulvar squamous cell carcinoma with a 5% probability [7, 8]. Standard therapy (first and second line) consists in the application of topic ultrapotent glucocorticoids or calcineurin inhibitors, which relieves the symptoms of the disease, prevents vulvar tissue fusion, and reduces the risk of malignant transformation [9, 10]. An adequate treatment requires a strict adherence to extended usage of this group of preparations, meanwhile a refractory course of VLS has been registered in some patients [11]. Although the first and second line therapy is effective, the mentioned preparations have serious local and systemic side-effects and in some cases the standard treatment fails to give desired results [12].

A new method of treatment using a fractional ablation with CO_2_ laser [13] has been proposed and gained a wide application in the world practice. The initial investigations have shown improvement of symptoms (especially dryness and dyspareunia), elasticity and vascularization of the vulvar skin [13, 14]. Later this therapy has demonstrated a better symptom control and greater satisfaction of patients with the treatment in comparison with the application of topical glucocorticosteroids [15]. Balchander and Nyirjesy have demonstrated effective therapy of glucocorticoid-resistant VLS with fractional CO_2_ laser in their study [16].

The principle of fractional CO_2_ laser action on biotissues consists in creation of micro-ablation (evaporation) zones, along the periphery of which areas of thermal tissue injury develop [17]. A therapeutic mechanism of action of the fractional CO_2_ laser in VLS has not been fully understood. It is supposed that microablations remove part of sclerotic tissue, while thermal injury of the boundary regions stimulates remodeling of the connective tissue through the production of the thermal shock proteins. Besides, the fibroblast proliferation and synthesis of new collagen and elastic fibers are enhanced, the number of blood vessels increases [18].

Despite the successful application of this treatment technique, no objective method to control therapy efficacy has yet been developed. Attempts have been undertaken to analyze histological changes of the vulvar tissue in response to the fractional CO_2_ laser therapy. Statistically significant increase of the dermis thickness and decrease of sclerotic zone have been registered as well as insignificant reduction of inflammatory infiltrate intensity a month after three procedures of laser treatment [19]. However, a histological examination cannot be recommended for a standard evaluation of therapy results or for monitoring the recurrence development due its invasiveness.

**The aim of the study** is to assess the possibility of non-invasive monitoring of vulvar skin structure recovery after treating vulvar lichen sclerosus with fractional CO_2_ laser using multi-modal optical coherence tomography.

Our previous investigations have demonstrated the ability of MM OCT to diagnose VLS intravitally even at the early stages, and also differentiate between various degrees of dermis lesions [20]. Besides, MM OCT allowed us to observe early histological signs of the VLS recurrence after photodynamic therapy, which makes it a promising method for non-invasive control of patients after treatment [21].

## Materials and Methods

### Patients and MM OCT examination

The study was carried out at the National Medical Research Center for Obstetrics, Gynecology, and Perinatology named after Academician V.I. Kulakov (Moscow, Russia) and was designed on the analysis of data from three clinical cases of histologically validated VLS. All patients were postmenopausal women who underwent an outpatient treatment with a fractional CO_2_ laser. The MM OCT examination was performed before a punch biopsy immediately before the first procedure of laser treatment and a month after a complete course of treatment (three procedures with an interval of 30– 40 days). The MM OCT examination was done at the point of the maximal visible tissue lesion on the labia minora, which corresponded to the site of biopsy. The zone of interest was marked with a medical marker and photographed. Two post-menopausal patients (aged 68 and 73 years) without vulva pathology were subject once to the MM OCT examination in the area of labia minora.

The MM OCT examination is completely painless and does not require anesthesia.

The study was approved by the local Ethical Committee of the National Medical Research Center for Obstetrics, Gynecology, and Perinatology named after Academician VI. Kulakov (protocol No.08 of September 21, 2023). Written informed consent to treatment and investigation was obtained from all patients.

### Treatment with fractional CO_2_ laser

Patients were treated with a surgical L’Med-1 CO_2_ laser apparatus (Russian Engineering Club, Tula, Russia; Certificate of Registration P3H 2014/1923). The vulvar skin was processes in a fractional ablation mode. The course consisted of three procedures with an interval of 30-40 days. The following parameters were used during the first procedure: super impulse duration — 900 us; filling density — 150 micro-areas per 1 cm^2^; number of impulses to a point — 1; the depth of irradiation penetration into the tissue — 1100 urn. The parameters used during the second and third procedures were as follows: super impulse duration — 900 us; filling density — 150 micro-areas per 1 cm^2^; the number of impulses into a point — 2; the depth of irradiation penetration into the tissue — 1400 urn. The size of the fractional scanning zone was 9x9 mm, the micro-area ablation diameter was 300 urn. The principle of choosing the parameters for fractional CO_2_ laser was described in more detail in the papers [22, 23].

Prior to laser treatment, a single shot sanitation of the vulva and vagina was performed by means of low-frequency ultrasound cavitation with an aqueous solution of chlorhexidine, followed by superficial anesthesia (prilocaine 25 mg + lidocaine 25 mg) applied to the vulvar skin with a 60-minute exposure. The treatment zone included the vulvar and perianal regions, the entire tissue area was processed once, the processed regions did not overlap each other.

### Multi-modal optical coherence tomography

In the present study we used a spectral multi-modal 1300-E OCT system (Biomedtech, Russia, Certificate of Registration OCP 2012/13479) equipped with a flexible fiber optic contact probe having a 5 cm length of the contact probe part and a 1 cm diameter. An articulated stand is used for precise positioning of the probe on the tissue.

The OCT system works at the central wavelength of 1310 nm with a 100 nm spectrum width. The visualization speed is 20,000 A-scans/s, depth resolution — 10 urn, lateral resolution — 15 urn. The OCT system creates a 3D dataset in 26 s, which consists of 256 pixels in depth (2 mm in air) and 512x512 pixels in the horizontal plane (3.4x3.4 mm).

**Figure 1.**
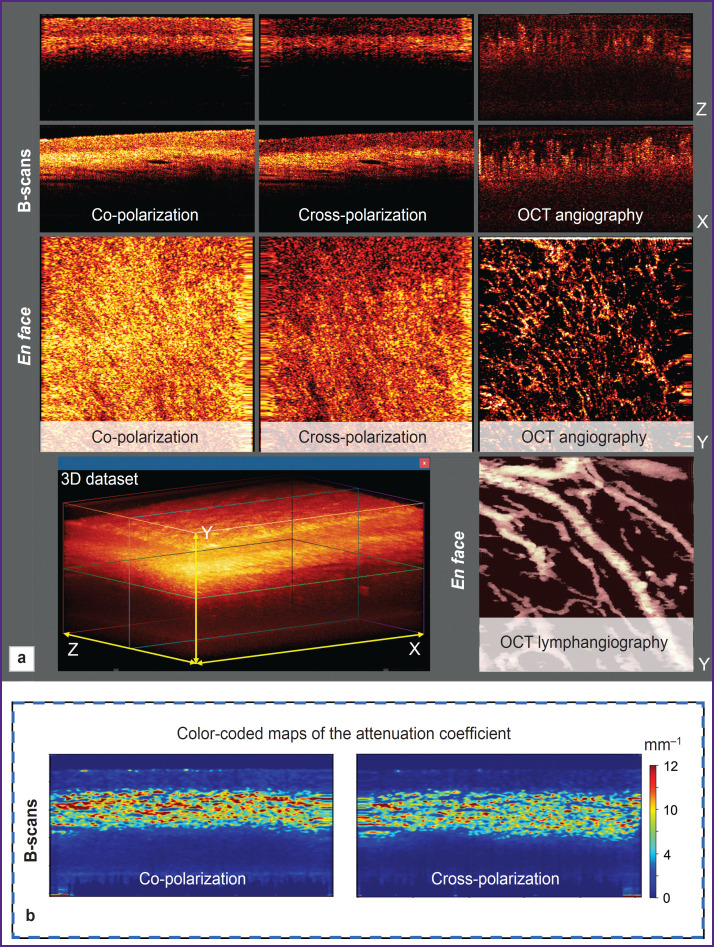
Modalities of the OCT examination: (a) the window of the program for viewing and recording MM OCT, demonstrates images of normal vulvar skin obtained in real time; X, Y, Z — coordinate planes of the OCT 3D dataset; (b) appropriate color-coded maps of the coefficient of OCT signal attenuation obtained as a result of its subsequent processing; the color-coded scale is on the right

Structural OCT images of the lateral section (B-scan) in co- and cross polarization, OCT angiographic image in *en face* projection, and OCT lymphangiographic images in the same *en face* projection are built in real time from the 3D dataset (Figure 1 (a)).

**Figure 2.**
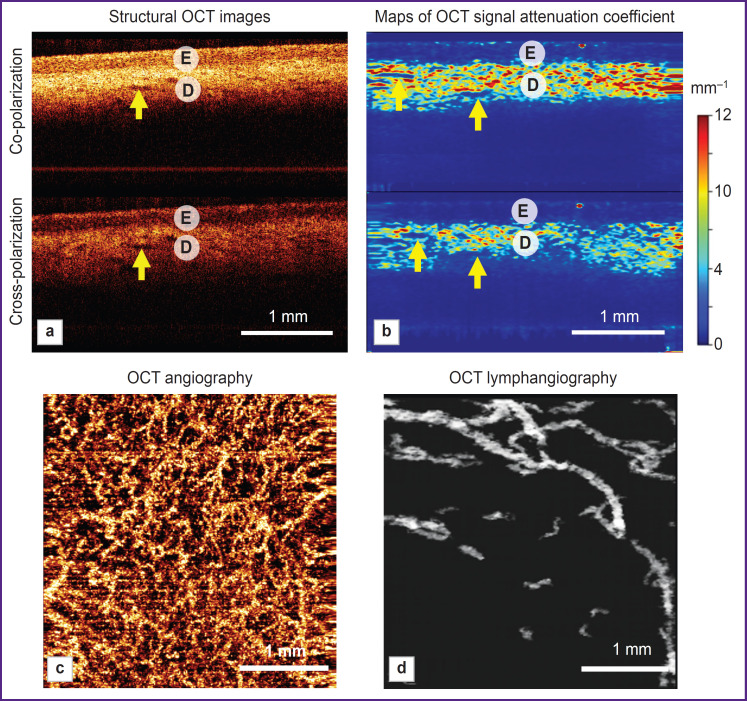
MM OCT examination of the normal vulvar skin. Patient K., 73 years, postmenopause: (a) structural OCT images in co- and cross-polarization (B-scan); (b) color-coded maps of the OCT signal attenuation coefficient in the co- and cross-channels (B-scan); (c) OCT angiographic *en face* image; (d) OCT lymphangiographic *en face* mages. E — epidermis, D — dermis, *arrows* — slit-like structures with a low signal correspond to lymphatic vessels

Structural images are presented as two conjugated B-scans: in co- and cross-polarizations (Figure 2 (a)). The OCT cross-channel is characterized by sensitivity to anisotropic (changing the direction of light polarization) structures such as collagen bundles of the dermis and keratinized layer (stratum corneum) of the epidermis (Figure 2 (b)).

OCT angiography is based on the assessment of variances of OCT signal speckles, which fixes the 3D picture of the tissue microcirculatory bed to the depth of the OCT signal penetration. The algorithm providing the compensation of probe movements as well as respiratory and cardiac movements of the patient is used. OCT lymphangiography employs the algorithm based on a 3D distribution of the OCT signal attenuation coefficient with the depth resolution. The obtained 3D vascular networks are presented in the 2D images in the projection of maximal intensity for blood vessels and moderate intensity for lymphatic vessels in the 0-350 urn depth range.

Additionally, we build color-coded maps of distribution of the OCT signal attenuation coefficient in the tissue in the B-scan projection (Figure 1 (b)). This presentation of the OCT signal attenuation coefficient intensifies contrast and improves visualization of tissue structures in comparison with structural OCT images. The calculation of this coefficient is an objective and standard approach, which describes the loss of the OCT signal with the depth due to dissipation and absorption of the probing irradiation [24].

### Visual evaluation of the MM OCT images

When evaluating the structural OCT images and maps of the OCT signal attenuation coefficient, the laminated structure of this organ formed by the epidermis and underlying fibrous connective tissue, dermis, is described. The following OCT features may be distinguished:

the thickness of the first layer (epidermis) may be both increased due to epidermis hypertrophy or acanthosis or reduced as the result of atrophy;

a high signal on the surface of the first layer (corresponds to the stratum corneum of the epidermis) is caused by hyperkeratosis (orthokeratosis or parakeratosis);

the boundary between the first (epidermis) and the second layer (dermis) may be contrast or less contrast. This parameter depends on the aggregate condition of the epidermis and dermis. The contrast is formed by a lower OCT signal level from the epidermis and a higher OCT signal level from the dermis (Figure 2 (a), (b)). The boundary contrast may be increased by enhancing the level of the OCT signal from the dermis (for example, due to a decreased number of lymphatic vessels in the dermis). The contrast may be reduced in several cases: by increasing the level of the OCT signal from the epidermis (for example, caused by hypergranulosis, i.e., an elevated accumulation of the keratogyalin granules in keratinocytes) or by decreasing the OCT signal from the dermis (due to homogenization of the collagen bundles and/or edema of the superficial dermis in VLS, and also due to the evident cellular inflammatory infiltrate);

the presence or a number of slit-like structures in the dermis with a low level of the OCT signal (correspond to lymphatic vessels), which may be reduced, for example, in VLS.

While evaluating angiographic and lymphangiographic OCT skin images (in the *en face* projection), one can visually assess the quantity and density of blood and lymphatic vessels in the dermis, their number in VLS is usually reduced relative to the norm.

### Quantitative evaluation of OCT data

The software developed by our team for the OCT data analysis allows us to obtain the quantitative values for the coefficient of OCT signal attenuation and the density of blood and lymphatic vessels at the specified depth of the 3D data volume.

To calculate the OCT signal attenuation coefficient, we applied the method with the depth resolution proposed by Vermeer [25] and modified by Moiseev [26]. The distribution of the attenuation coefficient values for each OCT dataset is presented in the form of color-coded maps (B-scans). Minimal and maximal values of the color-coded scale (red and blue, respectively) were chosen considering optimal contrast (0-12 mm^–1^). Quantitative values were computed for the entire dermis area and presented as a mean value ± standard deviation (for one frame).

The blood and lymphatic vessel density was quantitatively assessed using 2D maps built in the studied depth range (0-350 urn). The total area of all visualized vessels per unit of 2D map area was calculated and the diameter of the vessel lumen was computed as a doubled distance between the boundary of the vessel binary image and its skeleton. Vessels with overlapping binary boundaries and skeletons were assigned a thickness of 1 pixel [27]. For the lateral resolution of the applied OCT method, it corresponded to the vessel diameter below 15 um.

The epidermis thickness was calculated using color-coded maps for the coefficient of OCT signal attenuation by measuring from the epidermis surface to the dermis boundary in 5 regions, values are presented as a mean value ± standard deviation.

### Histological analysis

The histological investigation was performed before the treatment for diagnosing, evaluating the degree of dermis lesion in VLS, and verifying the obtained OCT data. The histological skin structure was evaluated by Van Gieson’s picrofuchsin staining: collagen fibers become crimson red, cell nuclei black, other tissue elements yellow. This staining is more informative than standard staining with hematoxylin and eosin in case of structural changes of the dermal collagen fibers. The histological preparations were analyzed using the automated EVOS M7000 imaging system (Thermo Fisher Scientific, USA).

## Results and Discussion

### MM OCT examination of vulvar skin in postmenopause without pathological changes

The structure of a normal vulvar tissue depends on the anatomical region and the menopause onset, which is important to consider when analyzing the MM OCT data. Depending on the localization, the normal vulvar tissue may be presented by the hair-bearing skin, hairless skin, or glycogenated mucous membrane. Previously, we demonstrated the MM OCT characteristic of the normal vulvar mucous membrane [28] and vulvar skin [20] with histological verification. The vulvar skin is known to be a hormone-dependent tissue, which is expected to result in structural changes after the menopause onset [29]. In this article, we consider the structure of the hairless skin of vulvar labia minora in postmenopausal period.

The hairless vulvar skin is characterized by lamination, which is observed in the structural OCT images and their respective maps of the OCT signal attenuation coefficient (Figure 2 (a), (b)). The epidermis has a 167±25 urn thickness. The boundary between the epidermis and dermis is contrast. The dermis has a high level of signal and high values of the attenuation coefficient in co- and cross-channels (5.9±1.7 and 3.1±1.2 mm^-1^, respectively). In the dermis there are slit-like structures with a low signal — lymphatic vessels (Figure 2 (a), (b), *arrows).* The OCT angiography demonstrates multiple blood vessels of various diameters (Figure 2 (c)), which are located in the tissue with high density ((3.7±0.3)-10^–4^ a.u.). The OCT lymphangiography shows lymphatic vessels of a larger diameter than the blood vessels (Figure 2 (d)), their density is (4.5±0.6).10^–2^ a.u.

### MM OCT and histological examination of the vulvar skin in lichen sclerosus

VLS changes the morphological structure of the vulvar tissue during the course of the disease. Commonly, early and late variants of VLS are distinguished clinically and histologically. The clinical division is based on structural changes of vulva anatomy, which occur at a late stage (narrowing of a vaginal opening, fusion or atrophy of the labia, clitoral hood sclerosis, and others) [30, 31]. The histological division is based on the evaluation of changes in the dermis, which result in its sclerosis (homogenization) at the late stage [32]. The histological classification takes into account the tissue microstructure and is suitable for interpretation of MM OCT data, but it does not always comply with clinical classification.

In this study, we consider three clinical cases, which were identified histologically as early, early with a marked dermal edema, and a late variant of VLS. In all cases, patients were postmenopausal.

#### Clinical case 1. Early vulvar lichen sclerosus (patient K., 72 years)

Brief clinical description: symptom duration — 1 month; complaints: intensive itching and burning in the region of labia majora and anus, which enhanced by the evening; on visual examination there were noted areas of whitening of vulvar skin and perianal region, excoriations.

**Figure 3.**
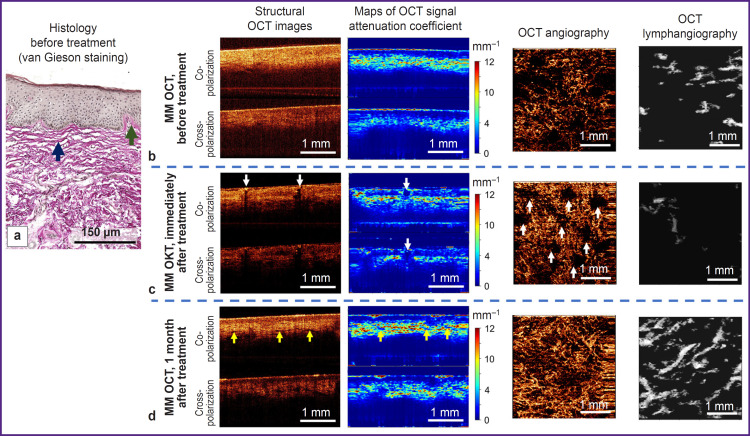
MM OCT and histological investigation of the skin affected by early vulvar lichen sclerosus (clinical case 1) before, immediately after, and 1 month after the treatment: (a)-(d): early vulvar lichen sclerosus. In the histological image, a *green arrow* shows focally thickened basement membrane, a *blue arrow*— lamellar fibrosis with edema between collagen bundles. In the MM OCT images, *yellow arrows* show slitlike structures in the dermis (corresponding to lymphatic vessels), *white arrows* — areas of microablation

Histological investigation demonstrates standard signs of early VLS. Epidermis has normal thickness, with orthokeratosis; the number of epidermal papillae is reduced, single basal keratinocytes are vacuolized. Focal thickening of the basement membrane is observed (Figure 3 (a), *green arrow).* Immediately under the epidermis there are long, wavy, thickened collagen bundles (Figure 3 (a), *blue arrow)*, spaces between the bundles are widened due to moderate edema. Signs of sclerotic zone formation are absent. A weak lichenoid lymphocytic inflammatory infiltrate is present.

On the MM OCT examination, significant changes in the tissue structure are observed relative to the norm (Figure 3 (b)). On the structural OCT images, the contrast of the boundary between epidermis and dermis is decreased both in co- and cross-channel. The maps of the attenuation coefficient are more informative, which makes it possible to observe a sufficiently contrast boundary between the epidermis and dermis and also a stratum corneum with a high value of attenuation coefficient. The epidermis thickness is decreased (127±28 urn). The attenuation coefficient of the dermis in the co-channel (3.5±1.6 mm^-1^) and cross-channel (2.6±1.2 mm^-1^) is reduced relative to the norm. Slit-like structures (lymphatic vessels) in the dermis are practically absent. The OCT lymphangiography shows single lymphatic vessels in the tissue (density (3.1±1.0)-10^-2^ a.u.). The OCT angiography reveals sharply decreased density of blood vessels in the tissue (1.3±0.5).10^–4^ a.u.).

#### Clinical case 2. Early vulvar lichen sclerosus with dermal edema (patient S., 59 years)

Brief clinical description: duration of the disease symptoms — 2 years. Complaints: long-existing itching in the region of vulva, onset of itching in the area of clitoris, dryness, and skin tightness. On visual examination: labia minora are deformed, reduced in size, areas of skin whitening are observed.

**Figure 4.**
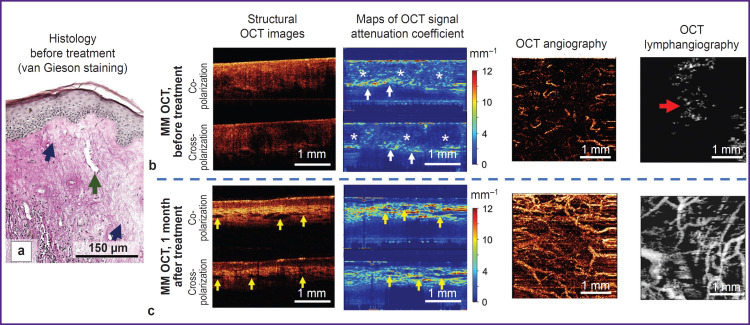
MM OCT and histological investigation of the skin affected by early vulvar lichen sclerosus with edema (clinical case 2) before treatment and 1 month after it: (a)-(c): early vulvar lichen sclerosus with a marked dermal edema. In the histological image, a *green arrow* shows a dilated blood vessel in the zone of dermal lesion; *blue arrows* — a boundary of edematous zone. In MM OCT images, *asterisks* show the zone corresponding to dermal edema; *white arrows* — the zone corresponding to preserved dermal skin; *yellow arrows* — slit-like structures in the dermis (correspond to lymphatic vessels); a *red arrow* points to the zone of edema on OCT angiography

The histological investigation demonstrates the picture typical of early VLS with dermal edema (Figure 4 (a)): atrophy of epidermis with orthokeratosis, the number of epidermal papillae are decreased. Degeneration of basal keratinocytes with their vacuolization and thickening of the basement membrane over the entire bioptate are observed. The superficial dermis consists of a large number of sufficiently thin collagen bundles, a marked edema of the dermis is observed (Figure 4 (a), *blue arrows).* Blood vessels in the lesion zone are single, their lumens are widened, walls thickened (Figure 4 (a), *green arrow).*

As compared to early VLS (clinical case 1), the MM OCT examination demonstrates the structural features associated with dermal edema. Thus, a zone of edema (Figure 4 (b), *asterisks)*, containing thin collagen bundles located with a low density, is visualized on the maps of the attenuation coefficient. The values of the attenuation coefficient are sharply reduced both in the co-channel (2.6±1.7 mm^–1^) and cross-channel (1.4±1.5 mm^–1^). Below the edematous zone there is a zone with preserved collagen fibers (Figure 4 (b), *white arrows)* and high values of the attenuation coefficient. On the lymphangiographic OCT images, edema is visualized as a “cloud” (Figure 4 (b), *red arrow).* Visualization of the edema in the lymphangiographic OCT images is connected with a weak light dissipation by the lymph and edematous liquid.

#### Clinical case 3. Late vulvar lichen sclerosus (patient G., 55 years)

Brief clinical description: duration of the disease symptoms — 4 years; complaints: moderate itching in the vulvar and perianal regions, vulva dryness; on visual examination: atrophy of labia minora and majora, fissures of the posterior commissure, whitening of the vulvar skin and perianal region in the form of a “keyhole"

**Figure 5.**
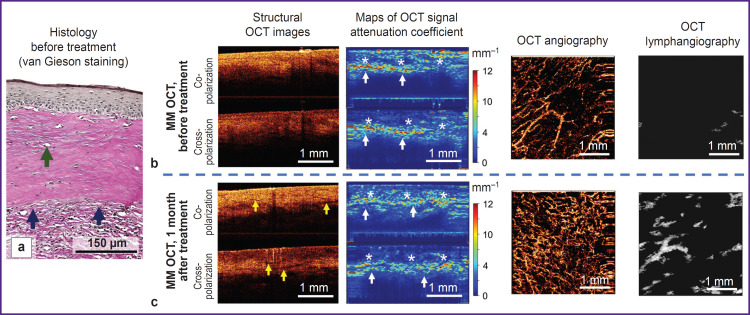
MM OCT and histological investigation of the skin affected by late vulvar lichen sclerosus (clinical case 3) before and 1 month after treatment: (a)-(c): late vulvar lichen sclerosus. In the histological image, a *green* arrow shows single widened blood vessels in the zone of dermis homogenization (sclerosis); *blue arrows* — a boundary between the zone of dermis homogenization (sclerosis) and the underlying preserved dermis. In MM OCT images, *asterisks* show the zone corresponding to homogenization (sclerosis) in the dermis; *white arrows* — the zone corresponding to preserved (fibrous) dermis; *yellow arrows* — slit-like structures in the dermis (corresponding to lymphatic vessels)

The histological investigation demonstrates classical pathognomonic picture for late VLS (Figure 5 (a)). A marked atrophy of the epidermis (lack of epidermal papillae), spongiosis, and vacuolization of basal keratinocytes are observed. Immediately under the epidermis there is a wide zone of homogenization of the collagen bundles — the zone of sclerosis (Figure 5 (a), *blue arrows).* Below the sclerotic zone, the dermis contains preserved collagen bundles and weakly expressed lymphocytic inflammatory infiltrate. Blood vessels in the sclerotic zone are single, dilated, with thickened walls.

As compared to early VLS (clinical case 1), the MM OCT examination demonstrates the features associated with formation of the sclerotic dermal zone. Thus, on the attenuation coefficient maps, the sclerotic zone (Figure 5 (b), *asterisks)*, has decreased values in the co-channel (3.3±1.4 mm^–1^) and the cross-channel (3.1 ±1.3 mm^–1^), which is connected with the loss of dissipating and anisotropic properties of the homogenized (sclerotic) dermis. Below the sclerotic tissue, there is the dermis with the preserved collagen bundles, which has high values of the attenuation coefficient and forms a clear contrast boundary with it (Figure 5 (b), *white arrows).*

### MM OCT control of the skin recovery 1 month after the course of treatment with fractional CO_2_ laser

Immediately after fractional laser exposure, regions of vulvar skin microablation (evaporation) may be observed in the structural OCT images and attenuation coefficient maps. The microablation regions possess a low OCT signal and low values of its attenuation coefficient (Figure 3 (c), *white arrows).* Their diameter is 285-300 urn. In the microablation regions of the OCT image there are no blood vessels, while between them increased density of blood vessels is observed ((2.4±0.5).10^–4^ a.u.) relative to the condition before the exposure ((1.3±0.5).10^–4^ a.u.). It is likely to be associated with vasodilatation and increased number of functioning vessels in the microcirculatory bed.

A month after the course of treatment with fractional CO_2_ laser, the MM OCT examination has revealed significant changes of the vulvar skin, which depended on the initial state of the tissue in VLS.

#### Clinical case 1. Early vulvar lichen sclerosus

Brief clinical description: the patient notes complete absence of symptoms; on visual examination the regions of skin whitening became less noticeable, the color of the skin looked more physiological.

The MM OCT examination has shown a more contrast boundary between the epidermis and dermis in the structural OCT images and the attenuation coefficient maps. The keratinizing layer remains, although it is less marked than before the treatment. The epidermis thickness is 184±27 urn. Slit-like structures (lymphatic vessels) appear in the dermis (Figure 3 (d), *yellow arrows).* The values of the attenuation coefficient in the dermis increase relative to the condition before the treatment both in the co-channel (4.9±1.8 mm^–1^) and cross-channel (2.8±1.4 mm^–1^) and are close to the values established for the normal skin, which is indicative of the dermal structure recovery. The OCT angiography and lymphangiography demonstrate a higher density of blood and lymphatic vessels in the tissue ((5.9±0.6).10^–4^ and (8.1±1.1).10^–2^ a.u., respectively), which is close to the values established for the normal vulvar skin (see the Table).

**Table:** Quantitative values of the data obtained by MM OCT examination of normal vulvar skin and the considered clinical cases*

Examinations	Epidermis thickness (μm)		Coefficient of OCT signal attenuation in co-channel (mm^–1^)		Coefficient of OCT signal attenuation in cross-channel (mm^–1^)		Density of blood vessels (×10^–4^ a.u.)		Density of lymphatic vessels (×10-^2^ a.u.)	
	Before therapy	After therapy	Before therapy	After therapy	Before therapy	After therapy	Before therapy	After therapy	Before therapy	After therapy
Early VLS (clinical case 1)	127±28	184±27	3.5±1.6	4.9±1.8	2.6±1.2	2.8±1.4	1.3±0.5	5.9±0.6	3.1±1.0	8.1±1.1
Early VLS with dermal edema (clinical case 2)	**	193±25	2.6±1.7	5.2±2.3	1.4±1.5	2.5±1.5	1.2±0.8	6.8±1.1	1.5±0.6	13.0±2.0
Late VLS (clinical case 3)	**	**	3.3±1.4	3.9±1.5	3.1±1.3	2.3±1.3	1.9±0.5	4.5±0.8	0.8±0.2	2.8±0.4
Norm, postmenopause	167±25		5.9±1.2		3.1±0.9		3.7±0.3		4.5±0.6	

* values are presented as mean ± standard deviation across the scanned region;

** it is impossible to reliably assess the epidermis thickness as the boundary between the epidermis and dermis is not contrast.

#### Clinical case 2. Early vulvar lichen sclerosus with dermal edema

Brief clinical description: the patient notes reduced itching of the vulva, no itching in the clitoris region, feeling of dryness and tightness of the vulva is absent. On visual examination: labia minora deformation is preserved although the skin color has a more physiological appearance.

The MM OCT picture of vulvar skin recovery complies completely with the clinical case 1 (Figure 4 (b); see the Table), there are no signs of dermal edema both in the attenuation coefficient maps and angiographic OCT images.

#### Clinical case 3. Late vulvar lichen sclerosus

Brief clinical description: the patient notes a slight reduction of itching of the vulva and perianal region, absence of dryness; on visual examination: atrophy of labia minora and majora remains, fissures of the posterior commissure are absent, whitening of the vulvar skin and perianal region is preserved.

The MM OCT examination has demonstrated that the contrast of dermis-epidermis boundary is not restored. The zone with low values in the co-channel (3.9±1.5 mm^–1^) and cross-channel (2.3±1.3 mnr^1^) corresponding to the dermal sclerotic zone is preserved in the attenuation coefficient maps. However, the OCT angiography and lymphangiography demonstrate the increasing density of blood and lymphatic vessels in the tissue ((4.5±0.8).10^–4^ and (2.8±0.4).10^–2^ a.u.) relative to the state before the treatment, which speaks of the revascularization of the sclerotic zone.

Thus, we have conducted for the first time the MM OCT evaluation of the qualitative and quantitative changes of the skin in early and late VLS before and 1 month after the three procedures of exposure to fractional CO_2_ laser, which has demonstrated that the result of vulvar skin recovery depends on the degree of vulva damage before treatment. In early VLS, when the lesion is concentrated in the region of dermal-epidermal junction, complete regeneration of the MM OCT skin structure up to its physiological coloration is observed. The recovery of attenuation coefficient values in co-and cross-channels, density of blood and lymphatic vessels to the level of the normal vulvar skin are noted. In late VLS with a vast sclerotic zone, the complete regeneration of the MM OCT vulvar skin structure is not observed. On visual examination, atrophy of vulvar structures is preserved, however, fissures have resolved, which is likely due to the improved tissue elasticity. The sclerotic zone still remains 1 month after the course of treatment, but at the same time, there is increased density of blood and lymphatic vessels in the dermis, which also reached the level of the normal skin.

### Limitations of the study

The drawback of the present study is a short term of observation of the skin recovery after treatment and small number of patients. However, a 1-month period is enough for regeneration of the skin after fractional CO_2_ laser therapy [33]. Further monitoring (after 3, 6, 12 months) should be carried out to control the VLS recurrence, as it has been demonstrated in our investigations exemplified by the photodynamic VLS therapy [21]. Continuation of the study will involve enlargement of the patient sample and application of the statistical analysis.

## Conclusion

The MM OCT vulva skin examination has demonstrated a positive effect of applying fractional CO_2_ laser for vulvar lichen sclerosus therapy on the epidermis thickness, the condition of collagen dermal bundles, and the density of blood and lymphatic vessel network. The result of skin regeneration after the treatment depended on the initial degree of the dermal lesion. For example, in early vulvar lichen sclerosus, all studied parameters reached the level typical of the normal vulvar skin, whereas in late VLS, the effect on the homogenized collagen bundles in the sclerotic zone was minimal, however, the density of the blood and lymphatic network increased. Thus, MM OCT may be proposed as a promising non-invasive method for monitoring skin regeneration after fractional CO_2_ laser treatment of vulvar lichen sclerosus.
